# Exploring uterine contractility frequency in infertile population: A comparative study among different control groups with and without a C-section defect

**DOI:** 10.5935/1518-0557.20240037

**Published:** 2024

**Authors:** Juan Carlos Castillo, Maria Martínez-Moya, Ana Fuentes, Belen Moliner, María Gonzalez, Andrea Bernabeu, Rafael Bernabeu

**Affiliations:** 1 Departament of Reproductive Medicine. Instituto Bernabeu. Alicante, Spain; 2 Catedra de Medicina Comunitaria y Salud Reproductiva. Universidad Miguel Hernandez. Elche, Spain

**Keywords:** Caesarean section, isthmocele, niche, uterine contractility, uterine peristalsis, ART

## Abstract

**Objective:**

Women undergoing IVF who have had a previous c-section (CS) have a lower live birth rate than those with a previous vaginal delivery. However, the precise underlying mechanisms need clarification. Does a previous CS affect the pattern of uterine contractility?.

**Methods:**

Prospective evaluation in patients undergoing frozen blastocyst embryo transfer in medicated endometrial preparation cycles. Twenty patients were included in groups: A/nulliparous. B/previous vaginal delivery. C/ previous CS without a niche, whereas fifteen patients were recruited in group D (CS and a niche). Patients employed estradiol compounds and 800 mg vaginal progesterone. A 3D-scan was performed the transfer-day where uterine contractility/minute was recorded.

**Results:**

Baseline characteristics (age, BMI, smoking, endometrial thickness) were similar. Mean frequency of uterine contractions/minute was similar between groups (1.15, 1.01, 0.92, and 1.21 for groups A, B, C, and D, respectively). There was a slight increase in the number of contractions in patients with a sonographic niche versus controls, not reaching statistical significance (*p*=0.48). No differences were observed when comparing patients with a previous C-section (regardless of the presence of a niche) to those without a C-section, either nulliparous (*p*=0.78) or with a previous vaginal delivery (*p*=0.80). The frequency of uterine contractions was similar between patients who achieved a clinical pregnancy and those who did not (1.19 vs. 1.02 UC/min, *p*=0.219, respectively).

**Conclusions:**

Our study found no significant difference in the frequency of uterine contractility between patients with or without a previous C-section or sonographic diagnosed niche. Further investigation is necessary to understand the physiological mechanisms affecting implantation in patients with isthmocele.

## INTRODUCTION

Caesarean section (CS) rates are rising worldwide. In Europe, one-in-four deliveries are by CS (25.7% in 2018) (Betran *et al*., 2021). However, there is no evidence that Caesarean rates higher than 10% are associated with reductions in maternal and foetal morbidity; hence, rising rates suggest increasing numbers of medically unnecessary, potentially harmful procedures. CS are associated with several obstetric and gynecologic potential complications. In addition, an adverse effect reported in recent studies is reduced subsequent fertility in general and ART population; in the latter, the presence of a CS defect (niche) seems to play a particular role (Diao *et al*., 2021). This data propounds the idea that the early implantation is affected by a previous CS especially in the presence of a niche (indentation) at the site of a C-section with a depth of at least 2mm; (Jordans *et al*., 2019). However, the precise underlying mechanisms are still pending to be clarified and it is uncertain whether the association is causal.

Recently, Vissers *et al.* (2020) postulated further hypotheses to explain the association between subfertility and the presence of a niche, and to define the knowledge gaps for future research perspectives. One of these hypotheses proponed a distorted contractility of the uterus (caused by fibrosis or interruption of the myometrial layer at the site of the CS/niche) as a potential physio-pathologic mechanism explaining the association with impaired fertility.

In the luteal phase of a natural cycle, uterine peristalsis is reduced to a minimum aiming to facilitate embryo implantation. *Contrario sensu*, a high uterine peristaltic frequency before embryo transfer has been correlated to lower clinical pregnancy rates in fresh and FET cycles (Zhu *et al*., 2014). With this background, our study aimed to investigate the frequency of uterine contractility as a potential explanation for the relationship between impaired fertility and C-section in the ART population.

## MATERIAL AND METHODS

### Study design and setting

Single center prospective cohort study carried out in the Department of Reproductive Medicine at Instituto Bernabeu in Alicante, Spain. The study was validated by the Instituto Bernabeu review committee (IBMR31/07-03-2022).

### Participants

Between September 2022 and January 2023, a total of seventy-five patients undergoing artificial endometrial preparation for blastocyst-stage FET cycles were included in the study. All patients were recipients from the oo-cyte-donation program undergoing their first or second FET cycle. Prior to participating, all individuals provided informed consent.

#### Inclusion criteria

- Endometrium thickness ≥7mm at the time of progesterone administration.
- Informed consent.
- Age between 18 and 51 years.
- Transfer of a single blastocyst-stage embryo (SET). The embryos were not tested for aneuploidy (PGT-A).
- Absence of endometrial fluid at the time of progesterone administration.
- Use of hormonal replacement therapy for endometrial preparation.

#### Exclusion criteria

- Polyps larger than 1cm.
- Fibroids larger than 3cm.
- Adenomyosis affecting the junctional zone.
- Uterine distortion caused by uterine malformations or fibroids invading the cavity.
- Previous myomectomy or isthmocele corrective surgery.
- Previous Asherman’s syndrome.
- Concurrent participation in another study that could alter the findings.

Twenty patients fulfilling the inclusion criteria were included in one of the following control groups:

- A: no previous delivery.
- B: a previous vaginal delivery.
- C: previous c-section without a niche.

**Figure 1:**
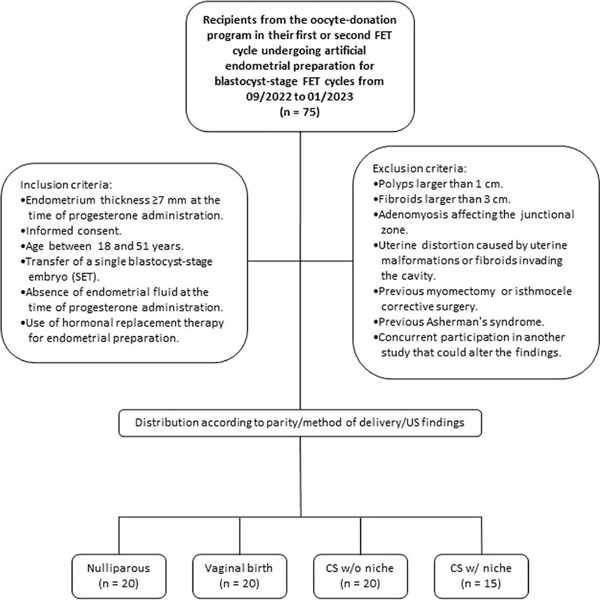
Flowchart of the study. FET: frozen embryo transfer. CS: Caesarean section with (w/) or without (w/o) a niche.

In the study group, 15 patients were included who had a previous c-section and sonographic diagnosed niche (Jordans *et al*., 2019) (Group D). Flowchart of the study in Figure 1.

### Study outcomes

The primary outcome investigated was the frequency of uterine peristalsis reported as contractions per minute, compared among the four groups.

Exploratory outcomes included the biochemical pregnancy rate per embryo transfer (a pregnancy diagnosed by the detection of beta hCG in serum or urine performed at least ten days after embryo transfer) and clinical pregnancy rate / embryo transfer (a pregnancy diagnosed by ultrasonographic visualization of a gestational sac) (Zegers-Hochschild *et al*., 2017).

### Endometrial preparation

To ensure standardization, all patients underwent a medicated cycle for endometrial preparation. Patients started treatment on day 1/2 of menstruation employing doses of oral estradiol (Progynova®, Bayer Hispania, Barcelona, Spain) —6 mg/day. After 10–14 days on estrogens, a vaginal 2D ultrasound was performed to measure endometrial thickness (at least 7 mm); then, 400 mg/12h vaginal progesterone (Cyclogest^®^ 400, Gedeon Richter Ibérica, S.A, Spain) was administered five days before ET and continued until the pregnancy test day and up the 12^th^ week in case of a positive test. Progesterone levels were measured on the day of embryo transfer and in the eventuality of “suboptimal” values (*i.e*. <9.2ng/ml) (Labarta *et al*., 2017), additional 25mg of s.c. progesterone (Prolutex®. IBSA S.L, Barcelona, Spain) was employed.

### Transvaginal ultrasound measurement

**Figure 2:**
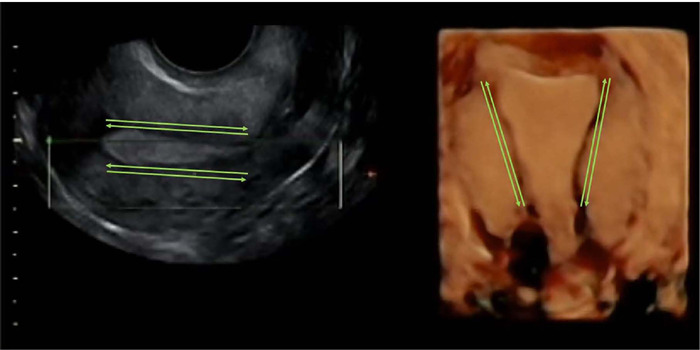
Two and three-dimensional ultrasound images of the uterus were obtained in the mid-sagittal section, focusing on the depiction of contractions in the longitudinal direction.

A 3D transvaginal ultrasonography scan (Voluson E10; General Electric) of uterine peristalsis was performed on the day of the transfer approximately 1 h before the procedure. After scanning the mid-sagittal plane of the uterus, the probe was fixed as steady as possible while a 6 min video of uterine peristalsis was recorded simultaneously. The records were analyzed at 15x accelerated frame speed calculating of uterine peristaltic waves. Frequency is reported as contractions per minute (Sammali *et al*., 2019) and the study focuses solely on the longitudinal direction of contractions (Figure 2). The scan evaluations were conducted by two operators with extensive experience (BM and MM). A comprehensive description of the image acquisition methodology can be found in our previously published video article (Moliner *et al*., 2021).

### Statistical methods

Considering the exploratory nature of the study and the limited existing literature on this topic, a formal sample size determination was not conducted. However, it is worth noting that functional studies of this kind often include a similar number of patients as reported in our study (van Gestel *et al*., 2003; Rees *et al*., 2023).

Statistical analysis was performed using Rstudio desktop. A comparative mean study was conducted using ANOVA test after verifying the normality of the data in the groups (BMI, age, and endometrial thickness) using the Shapiro test. Contraction frequency was expressed as a mean. Normal distribution was assessed in all groups (Shapiro-Wilk normality test). Based on the results, either a non-parametric (Wilcoxon rank sum test) or a parametric test (t-student) was applied for statistical analysis.

## RESULTS

### Patients characteristics

Table 1 shows that patients did not differ in baseline characteristics, including age, BMI, smoking status, endo-metrial thickness prior to the addition of progesterone and progesterone levels on the day of the embryo transfer.

**Table 1:** Baseline characteristics.

Variable	Group A (n=20)	Group B (n=20)	Group C (n=20)	Group D (n=15)	*p*-value*
BMI	23.15±3.18	25.25±5.62	23.86±3.43	23.46±3,77	0.8
Age	40.3±6.2	41.16±5.91	42.85±3.55	41.33±4.40	0.3
Endometrial thickness	8.67±1.73	9.02±1.01	9.01±1.16	9.23±2.09	0.14
Progesterone levels	17.47±10.16	14.44±7.08	18.58±8.15	16.84±8.32	0.37

Values are presented as mean ± SD, unless otherwise stated

*ANOVA test

### Contraction frequency

**Figure 3:**
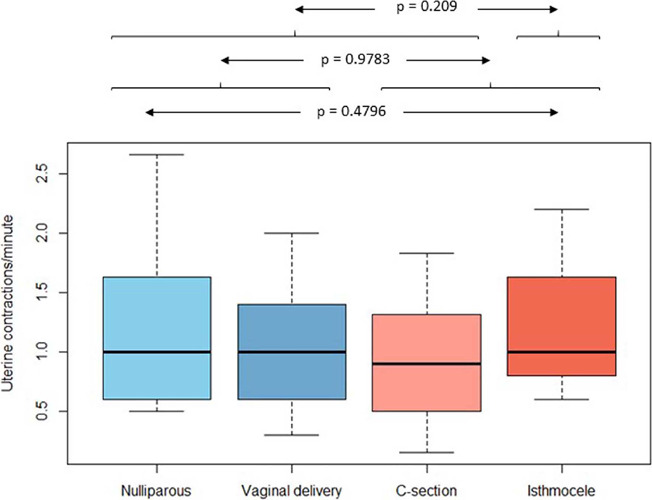
Box plot analysis. No significant difference in the comparison of all four groups, as indicated by a *p*-value of 0.4796. Similarly, no distinction was observed between the groups with and without previous C-section (*p*=0.9783) or between patients with sonographic diagnosed niche compared to the others (*p*=0.209).

The mean number of contractions per minute was comparable across all groups [1.15, 1.01, 0.92 and 1.21 (groups A, B, C and D; respectively)], with no statistical significance found. There was also no difference in contraction frequency between patients with a previous C-section and those without (1.05 *vs*. 1.08; *p*=0.978) Figure 3. Although there was a slight increase in mean number of contractions in patients with a sonographic diagnosed niche compared to those with a previous vaginal delivery, this difference was not statistically significant (1.21 *vs*. 1.01; *p*=0.26).

### Additional exploratory reproductive outcomes

Table 2 presents the reproductive outcomes within the studied population. No significant differences were found in the rates of biochemical or clinical pregnancy among groups. Furthermore, the frequency of uterine contractions was similar between patients who achieved a clinical pregnancy and those who did not. (1.19 *vs*. 1.02 UC/min, *p*=0.219, respectively). In the analysis comparing different groups, the occurrence of contractions showed similar frequencies in patients who achieved a clinical pregnancy and those who did not in groups A, B, and C. However, in group D specifically, patients who achieved a clinical pregnancy had a higher frequency of uterine contractions. (1.89 *vs*. 1.02 UC/min, 95%CI (-1.4, -0.50), *p*>0.009) (Table 3).

**Table 2:** Reproductive outcomes.

Variable / embryo transfer	Group A (n=19)**	Group B (n=20)	Group C (n=20)	Group D (n=15)
Biochemical pregnancy rate % (n)	63.16 (12)	30 (6)	35 (7)	26.67 (4)
Early pregnancy loss rate % (n)	26.32 (5)	10 (2)	5 (1)	6.66 (1)
Ongoing pregnancy rate % (n)	36.84 (7)	35 (5)	0 (6)	20 (3)

Values are presented as percentage (%) and number, unless otherwise stated

**One patient could not undergo embryo transfer due to an improper thawing

**Table 3:** Uterine contractility rate according to pregnancy outcomes.

Variable	Group A	Group B	Group C	Group D
Ongoing pregnancy	1.07	0.92	1.07	1.89
No gestation	1.21	1.03	1.02	1.02
*(CI)*, *p* -value*	*(-0.5, 0.80), 0.9*	*(-0.4, 0.69), 0.68*	*(-0.52, 0.42), 0.81*	*(-1.4, -0.50), 0.009*

Values are presented as mean of uterine contractions/minute, unless otherwise stated

CI: confidence interval

*Wilcoxon test

## DISCUSSION

The uterine incision in a Caesarean section might compromise the integrity of the junctional zone leading (in theory) to poor contractility of the uterine muscle around the scar; this phenomenon may be even more prominent in the presence of a CS defect. This assumption is reinforced by observations describing a significant decrease in muscular density in the myometrium surrounding a niche. However, the results of our exploratory study do not suggest any indications of distorted uterine contractility during blastocyst stage frozen embryo transfer in artificially prepared endometrial cycles among infertile patients with a previous C-section. Additionally, our findings revealed that the frequency of uterine contractions measured on the day of frozen blastocyst transfer remained similar regardless of the presence of a C-section or uterine niche. Nonetheless, -although not statistically significant-, patients with a sonographic diagnosed niche displayed a higher frequency of contractions (1.21 *vs*. 1.01) compared to those with a previous vaginal delivery. This suggests that decreased fertility following a C-section may be due to various factors other than an altered uterine contractility pattern, but further research, particularly within the subgroup of patients with a niche, is necessary to fully understand and confirm these observations.

As recently published, when explored in artificial cycles our transvaginal ultrasound methodology was able to identify significant differences in uterine contractility and progesterone levels between the hypercontractility and normal contractility groups, which suggests that our methodology is robust and effective (Moliner *et al*., 2021). Performing the evaluation of uterine contractions in a hormonal replacement cycle has the advantage of exposing all patients to similar standardized protocol conditions; but limits extrapolation to other scenarios of endometrial environment such as fresh-transfer IVF cycles or other types of endometrial preparation for FET (*i.e.* natural or stimulated cycles).

The primary limitation of the presented results is the small sample size of the study population. Nonetheless, the validity of the findings is supported by their overall consistency with the currently recognized patterns of uterine peristalsis observed throughout the menstrual cycle as recently published (Rees *et al*., 2023). Our limited sample size prevents us from making definitive conclusions. However, we believe that if there were a physiological change in contractility frequency following a C-section, it may have been noticeable in the analysis of the 35 patients in the C-section group. As an additional limitation, it is important to note that the analysis conducted in this study did not take into account other aspects of uterine dynamics, including factors such as amplitude, width, or direction of contractions, as well as characteristics of the uterine niche, such as size, presence of ramifications, or residual endometrial thickness. These aspects require further investigation, but, to the best of our knowledge, specific characteristics of the uterine niche have not been correlated with adverse pregnancy outcomes in ART population.

While reproductive outcome data is provided, it is important to exercise caution when interpreting these results due to the limited sample size analyzed and the potential for increased risk of type II error. Overall, the presence of a cesarean section did not appear to affect reproductive outcomes in our dataset. Additionally, the rate of uterine contractions was comparable between pregnant and non-pregnant patients, except in group D where women who achieved a clinical pregnancy exhibited a higher frequency of uterine contractions (1.89 UC/min) compared to non-pregnant counterparts (1.02 UC/min). Notably, this mean value of 1.89 UC/min aligns with the range of uterine peristalsis associated with successful pregnancies in artificial cycles for frozen embryo transfer, as illustrated in Zhu et al.’s paper (Zhu *et al*., 2014). The study found that the clinical pregnancy rate was highest when no more than 2.0 waves/min were observed before embryo transfer, declining with increasing wave frequency, and experiencing a significant decrease when >3.0 waves/min were observed.

## CONCLUSION

Our findings indicate that the frequency of uterine contractions on the day of frozen blastocyst transfer remained consistent, irrespective of the presence of a C-section or uterine niche. The decreased fertility observed following a C-section may be attributed to various factors other than an altered uterine frequency wave pattern. Further research with larger sample sizes is needed to validate our findings and to better understand the underlying mechanisms behind post-C-section subfertility.
